# ^68^Ga PSMA-11 PET with CT urography protocol in the initial staging and biochemical relapse of prostate cancer

**DOI:** 10.1186/s40644-017-0133-5

**Published:** 2017-12-21

**Authors:** Amir Iravani, Michael S. Hofman, Tony Mulcahy, Scott Williams, Declan Murphy, Bimal K. Parameswaran, Rodney J. Hicks

**Affiliations:** 1 Centre for Molecular Imaging, Department of Cancer Imaging, Peter MacCallum Cancer Centre, 305 Grattan Street, Melbourne, Australia; 2 Sir Peter MacCallum Department of Oncology, University of Melbourne, 305 Grattan Street, Melbourne, Australia; 3 Sir Peter MacCallum Department of Radiation Oncology, University of Melbourne, 305 Grattan Street, Melbourne, Australia; 4 Sir Peter MacCallum Department of Surgical Oncology, University of Melbourne, 305 Grattan Street, Melbourne, Australia

**Keywords:** PSMA PET/CT, PSMA-HBED PET, ^68^Ga-PSMA-11 PET, CT urogram, Prostate cancer, Biochemical recurrence

## Abstract

**Background:**

^68^Ga-labelled prostate specific membrane antigen (PSMA) ligand PET/CT is a promising modality in primary staging (PS) and biochemical relapse (BCR) of prostate cancer (PC). However, pelvic nodes or local recurrences can be difficult to differentiate from radioactive urine. CT urography (CT-U) is an established method, which allows assessment of urological malignancies. The study presents a novel protocol of ^68^Ga-PSMA-11 PET/CT-U in PS and BCR of PC.

**Methods:**

A retrospective review of PSMA PET/CT-U preformed on 57 consecutive patients with prostate cancer. Fifty mL of IV contrast was administered 10 min (range 8–15) before the CT component of a combined PET/CT study, acquired approximately 60 min (range 40–85) after administration of 166 MBq (range 91–246) of ^68^Ga-PSMA-11. PET and PET/CT-U were reviewed by two nuclear medicine physicians and CT-U by a radiologist. First, PET images were reviewed independently followed by PET/CT-U images. Foci of activity which could not unequivocally be assessed as disease or urinary activity were recorded. PET/CT-U was considered of potential benefit in final interpretation when the equivocal focal activity in PET images corresponded to opacified ureter, bladder, prostate bed, seminal vesicles, or urethra. Student’s T test and Pearson’s correlation coefficient was used for assessment of variables including lymph node size and standardized uptake value.

**Results:**

Overall 50 PSMA PET/CT-U studies were performed for BCR and 7 for PS. Median PSA with BCR and PS were 2.0 ± 11.4 ng/ml (0.06–57.3 ng/ml) and 18 ± 35.3 ng/ml (6.8–100 ng/ml), respectively. The median Gleason-score for both groups was 7 (range 6–10). In BCR group, PSMA PET was reported positive in 36 (72%) patients, CT-U in 11(22%) patients and PET/CT-U in 33 (66%) patients. In PS group, PSMA PET detected the primary site in all seven patients, of which one patient with metastatic nodal disease had negative CT finding. Of 40 equivocal foci (27/57 patients) on PET, 11 foci (10/57 patients, 17.5%) were localized to enhanced urine on PET/CT-U, hence considered of potential benefit in interpretation. Of those, 3 foci (3 patients) were solitary sites of activity on PSMA imaging including two local and one nodal site and 4 foci (3 patients) were in different nodal fields.

**Conclusions:**

PET/CT-U protocol is a practical approach and may assist in interpretation of ^68^Ga-PSMA-11 imaging by delineation of the contrast opacified genitourinary system and matching focal PSMA activity with urinary contrast.

**Electronic supplementary material:**

The online version of this article (10.1186/s40644-017-0133-5) contains supplementary material, which is available to authorized users.

## Background

Worldwide, prostate cancer (PC) is the most common malignancy in men [1]. Accurate staging of PC is of high importance for patient management as treatment selection is mainly influenced by the presence or absence of metastases [2]. Of all patients with PC undergoing therapeutic regimens with curative intent, about a quarter will ultimately have BCR with rise in PSA level. Of those, 50% will have local recurrence and 50% systemic disease with or without local recurrence [3, 4]. For an optimized and timely management, accurate primary staging (PS) of the disease and location of recurrence is essential. But at low PSA levels, small lymph nodes below the size threshold are commonly missed by conventional imaging [5]. The European Association of Urology (EAU) advocates bone scan and abdominopelvic CT only for patients with BCR after radical prostatectomy(RP) who have PSA level of higher than 10 ng/ml or PSA doubling time less than six months or bone pain [6]. Therefore, salvage radiotherapy (SRT) after RP is commonly decided on the basis of BCR, without imaging [6].To date, early and accurate localization of BCR remains a major challenge for all conventional imaging methods [7–9].

Prostate-specific membrane antigen (PSMA) is a type II membrane glycoprotein that is highly expressed by many PCs and correlates with tumor aggressiveness, metastatic and recurrent disease [10]. Positron Emission Tomography (PET)/CT imaging using Glu-NH-CO-NH-Lys-(Ahx)-[^68^Ga(HBED-CC)] (^68^Ga-PSMA-11) as a ^68^Ga-labelled PSMA ligand can detect PC metastases and relapses and with high contrast and specificity by binding to the extracellular domain of PSMA [11, 12]. The sensitivity and specificity of ^68^Ga-PSMA-11 PET/CT for detection of nodal metastases in PS is 75% and 96%, respectively and, both 86% in BCR [13, 14]. However, ^68^Ga-PSMA-11 PET/CT is limited by intense radiotracer activity in the kidneys, ureters and urinary bladder due to urinary excretion [12]. Ureters also not infrequently have abrupt turns and kinks throughout the course with associated urinary pooling, particularly as they cross major vascular structures. Bladder diverticulae, postsurgical resection cavities, post-instrumentation urethral dilatation and refluxed urine into the ejaculatory duct or retained seminal vesicles following prostatectomy can also retain urine. Excreted radioactivity in the urine can be difficult to differentiate from small pelvic nodes or local recurrence lying close to these structures. Acquisition of contrast-enhanced CT in the urogram phase as part of PET/CT protocol may potentially overcome this problem by distinguishing urinary activity from radiotracer activity. In this manuscript we present our experience and diagnostic utility of incorporating CT urography (CT-U) protocol in ^68^Ga-PSMA-11 PET imaging in primary staging (PS) and BCR of PC.

## Methods

### Patients

This study is a retrospective analysis of ^68^Ga-PSMA-11 PET/CT on consecutive patients referred to our center from July 2015 to October 2015 with histopathological diagnosis of PC after introduction of this CT methodology as a standard of care in patients with adequate renal function and without prior contrast allergy. Overall, 57 patients were included in the study. Of those, 50 patients with mean age of 64 years (range 47–78) were referred with BCR following prostatectomy, external beam radiation, brachytherapy or combination of these administered with curative intent. Seven patients with newly diagnosed PC were referred for initial staging prior to curative intent treatment with mean age of 64 years (range 58–78). This research has been approved by the institutional ethics committee and patient consent was waived (approval number: 15/46R). All investigations were performed as part of routine clinical care.

### Image acquisition and protocol

For PET, 2 MBq/kg of ^68^Ga-PSMA-11 were injected intravenously (mean 166 MBq, range 91–246 MBq). This was followed by an uptake period of mean 62 min (range 40–85 min). Using the same intravenous line, 50 ml of Omnipaque 300 mg/ml contrast medium (GE Healthcare, Princeton, NJ) was administered a mean of 10 min (range 8–15) minutes prior to CT acquisition in order to obtain a pyelogram phase CT. The conventional unenhanced or nephrographic phases of CT-U [15] were not acquired. Renal function was assessed by estimated glomerular filtration rate (eGFR) on peripheral blood test prior to contrast injection. Prior history of allergic reaction to intravenous iodine contrast was sought. Oral hydration was encouraged during uptake time. Patients were asked to void before imaging. Sequential CT and PET acquisition was then performed on an integrated PET/CT device (GE Discovery PET/CT 690, GE Healthcare, Milwaukee, WI or Siemens Biograph 16 PET/CT, Siemens Healthcare, Erlangen, Germany). The CT portion of the study was acquired in cranio-caudal direction encompassing vertex to mid-thigh with patient supine with slice thickness of 5 mm, increment of 1.5 mm, 140 keV, 220 mAs and 0.6 pitch. PET acquisition was subsequently acquired in caudo-cranial direction to minimize mis-registration in the pelvis as the primary region of interest. 3-D acquisition was performed with emission data corrected for randoms, scatter and decay. Reconstruction was conducted with an ordered subset expectation maximization (OSEM) algorithm with 2 iterations/8 subsets and Gauss-filtered to a transaxial resolution of 5 mm at full-width at half-maximum (FWHM). Attenuation correction was performed using above mentioned CT-U data. PET and CT-U were performed using the same protocol for every patient on both cameras.

### Image analysis

Image analysis was performed using an appropriate workstation and software (Syngo MMWP VE31A, TrueD, Siemens, Berlin, Germany) for the stand-alone CT assessment and (MIM 5.4.4; MIM Software, Cleveland, OH) for the combined PET/CT study. Two nuclear physicians, with prior experience of more than 1000 PSMA PET/CT, reviewed PET and PET/CT-U data and reached consensus on cases. First, PET images (three planes and MIP image) were reviewed and the number of focal (not linear or curvilinear) abnormalities, which could not unequivocally be determined to represent urinary activity was recorded. These were regarded as suspicious for prostate cancer. Then PET/CT-U images were reviewed and number of equivocal foci which corresponded to the contrast-enhanced urine were recorded.

**Fig. 1:**
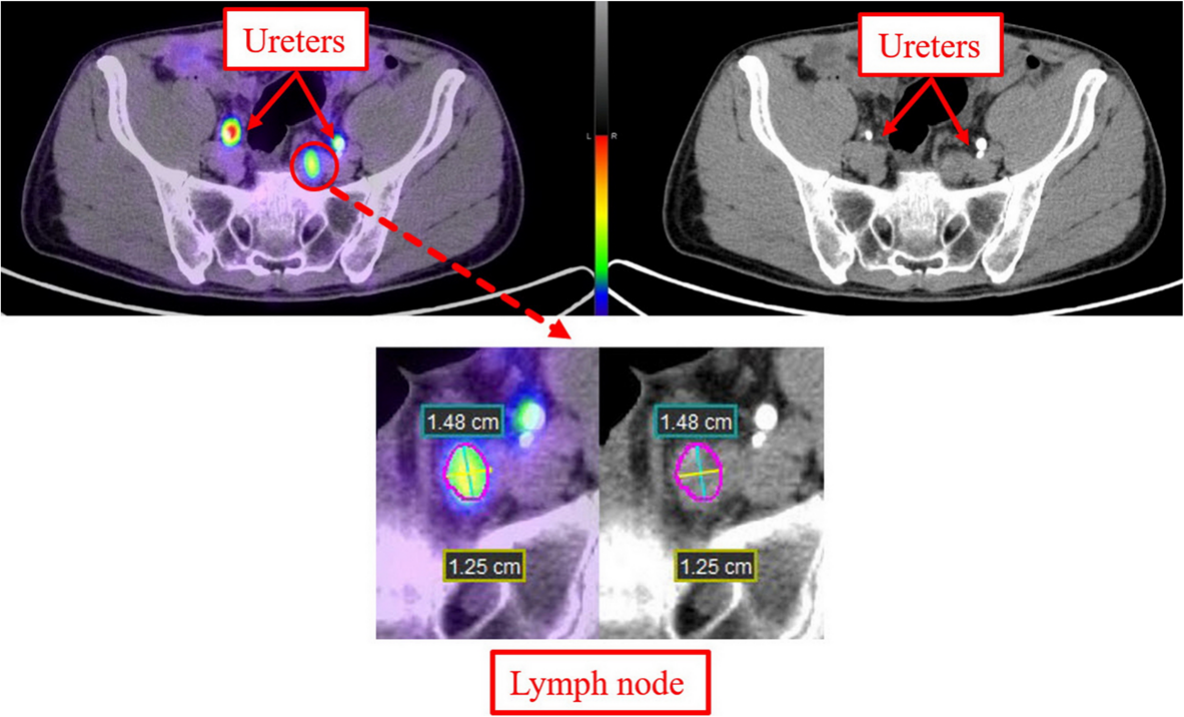
Top row. PET/CT-U and CT-U images show PSMA activity corresponding to opacified ureters and a left pelvic lymph node. The high-density focus posterior to the left ureter is a phlebolith in the iliac vessel. Bottom row. Magnified PET/CT-U and CT-U with semi-automatic PET edge detection with lymph node measurements

Maximum standardized uptake values (SUVmax) were calculated from manually drawn regions of interest over the sites of focal increased uptake including in the prostate, lymph nodes, ureters at the same level as any identified lymph nodes or distant metastatic sites. Short-axis, long-axis and volume of each positive PSMA lymph node was measured on co-registered PET/CT-U images semi-automatically by using PET edge tool of MIM software (MIM 5.4.4; MIM Software, Cleveland, OH), which was based on tumor gradient for segmentation. Distance between the opacified ureter and the ipsilateral lymph node was measured to assess the proximity of these foci (fig.1).

One radiologist reviewed the standalone CT-U images independently. Number and size of lymph nodes and site of local recurrence was recorded. Criteria for metastatic lymph nodes on CT-U was short axis larger than 8 mm. An osseous or soft tissue lesion which did not have all characteristics of a PC metastasis, was considered equivocal. These were considered negative for the final statistical analysis. Based on length of contrast opacified ureters, four groups were defined as follows; proximal ureters from renal pelvis to common iliac bifurcation, distal ureters from common iliac bifurcation to the vesicoureteral junction, entire length of both ureters, and patchy/unilateral ureter.

### Statistical analysis

All results were expressed as mean ± SD. Student’s T test was used to assess the difference between the mean values. Pearson correlation coefficients (r) were measured. For statistical analysis Excel software was used (Microsoft Office 2013, Redmond, WA). A *p* value lower than 0.05 was considered statistically significant.

## Results

Overall, 50 ^68^Ga-PSMA-11 PET/CT-U studies were performed for BCR and 7 for PS of PC. The median PSA value for patients with BCR and PS was 2.0 ± 11.4 ng/ml (0.06–57.3 ng/ml) and 18 ± 35.3 ng/ml (6.8–100 ng/ml), respectively. The most common GS for both groups was 7 (range 6–10).

### ^68^Ga-PSMA-11 PET, PET/CT and CT-U

**Fig. 2:**
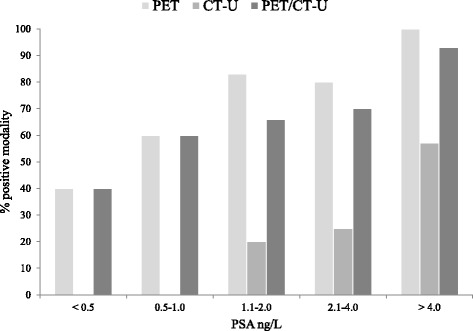
Percentage of positive result of each modality stratified by PSA level demonstrates an increasing likelihood of a positive imaging result with increasing PSA level. Most discordant cases between CT and PSMA PET positivity were in patients with a PSA <4ng/L

Overall in BCR group, PSMA PET alone was reported positive in 36 patients (72%), CT-U in 11 patients (22%), PSMA PET/CT-U in 33 patients (66%) and in 36 patients (72%) at least one of the above modalities. Figure 2 shows the proportion of positive results of each modality stratified by PSA level. Patients’ characteristics and modality based results are presented in Additional file 1: Table S3 and S4.

PSMA PET detected PC in all seven patients for PS, which was confirmed either by histopathology or MRI. PSMA PET and PSMA PET/CT-U detected nodal metastasis in one patient while CT-U was negative in all seven patients. The mean SUVmax of prostate lesions was 14 ± 7.5 (range 7–26.1).

**Fig. 3:**
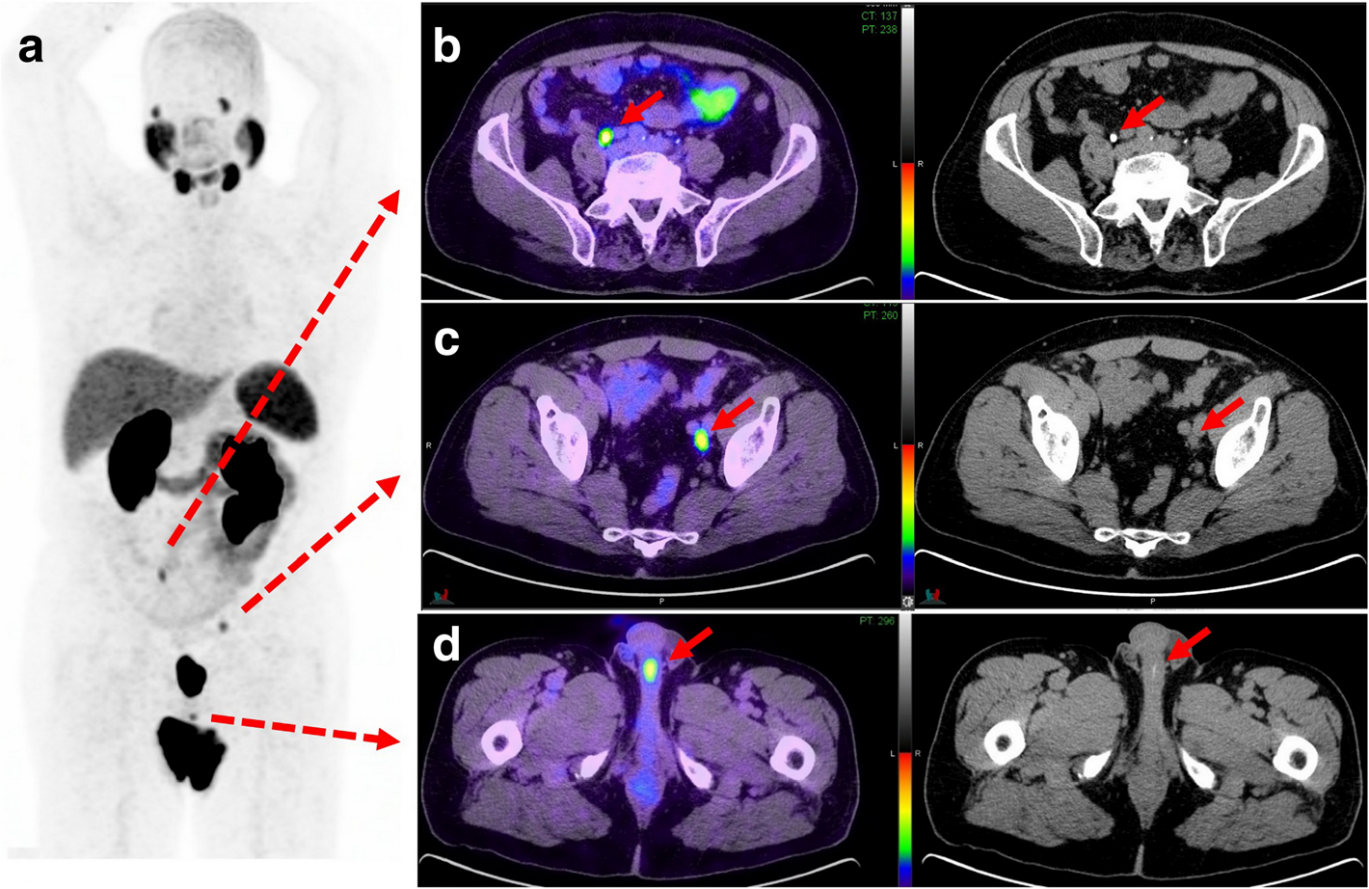
Sixty-six-year-old patient with BCR and PSA of 1.5 ng/L. Panel **a**. PSMA PET maximum intensity projection (MIP) image shows three foci of activity. Panel **b**. PET/CT-U and CT-U axial images shows focal activity corresponding to opacified right ureter (arrows). Panel **c**. PET/CT-U and CT-U axial images show focal uptake corresponding to a left pelvic lymph node (arrows). Absence of urinary opacification excluded the urinary pooling as potential explanation for this focal activity and this focus was reported as nodal metastasis. Panel **d**. PET/CT-U and CT-U axial images show focal activity corresponding to contrast agent in the urethra (arrows)

**Fig. 4:**
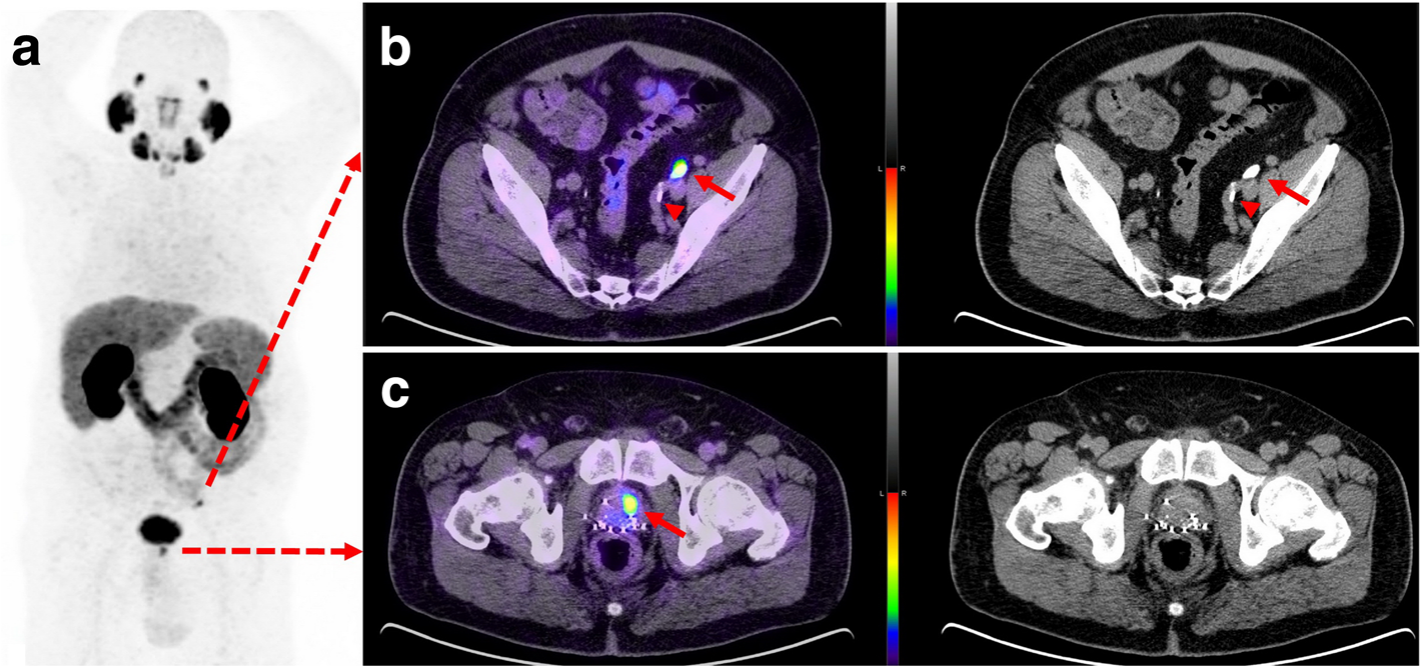
Sixty-six-year-old man with BCR and PSA 3.2 ng/L following brachytherapy. Panel **a**. PSMA PET MIP image demonstrates two foci of activity. Panel **b**. PET/CT-U and CT-U axial images show focal activity in the left pelvis corresponding to opacified, ectatic ureter (arrows). High density focus more posteriorly relates to a calcified plaque in the iliac vessel (arrow head). Panel **c**. PET/CT-U and CT-U axial images show focal uptake in the left side of prostate, consistent with local recurrence

**Fig. 5:**
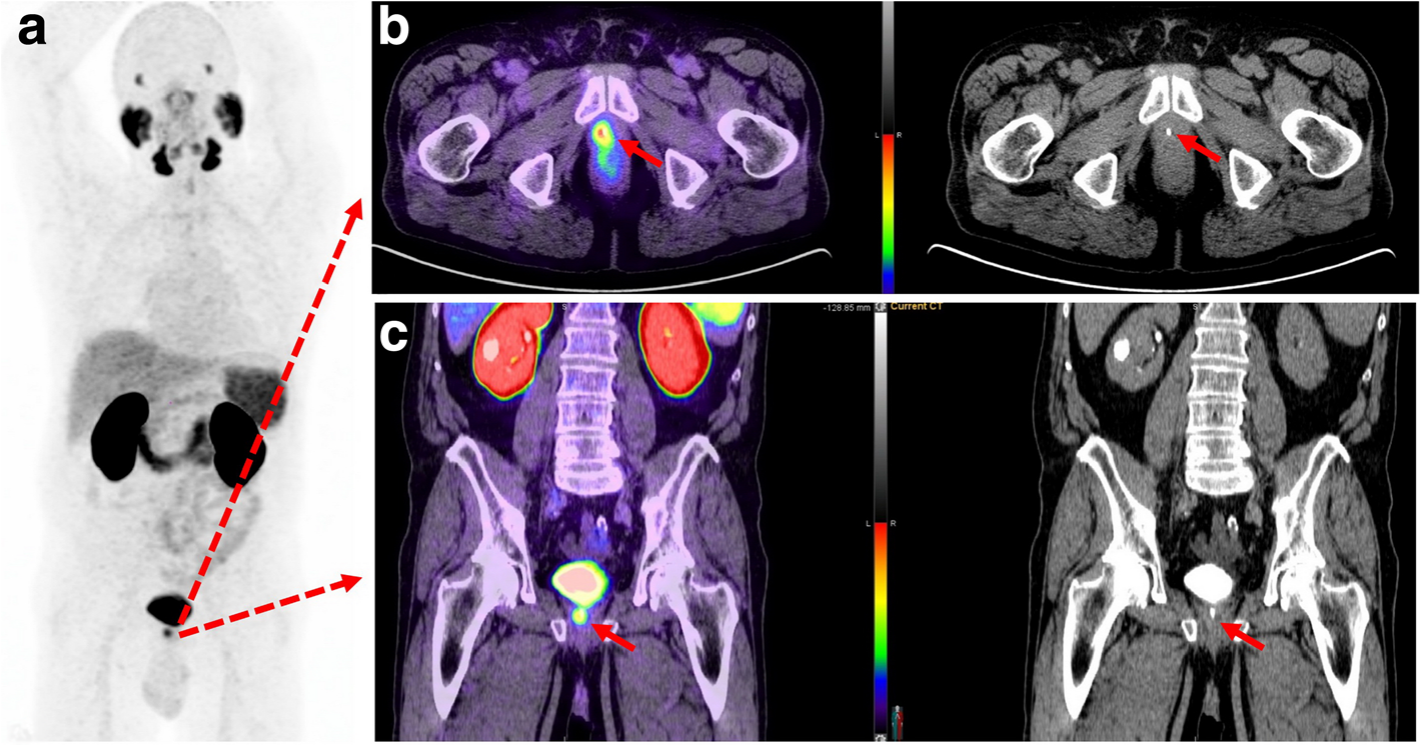
Seventy-six-year-old man with BCR and PSA 5.7 ng/L following prostatectomy. Panel **a**. PSMA PET MIP image shows focal activity in the prostatectomy fossa. Panel **b**, **c**. PET/CT-U and CT-U axial (top row) and coronal (bottom row) show focal activity corresponding to contrast material in the urethra-vesical junction. No PSMA-avid site of recurrence was detected in this patient

**Fig. 6:**
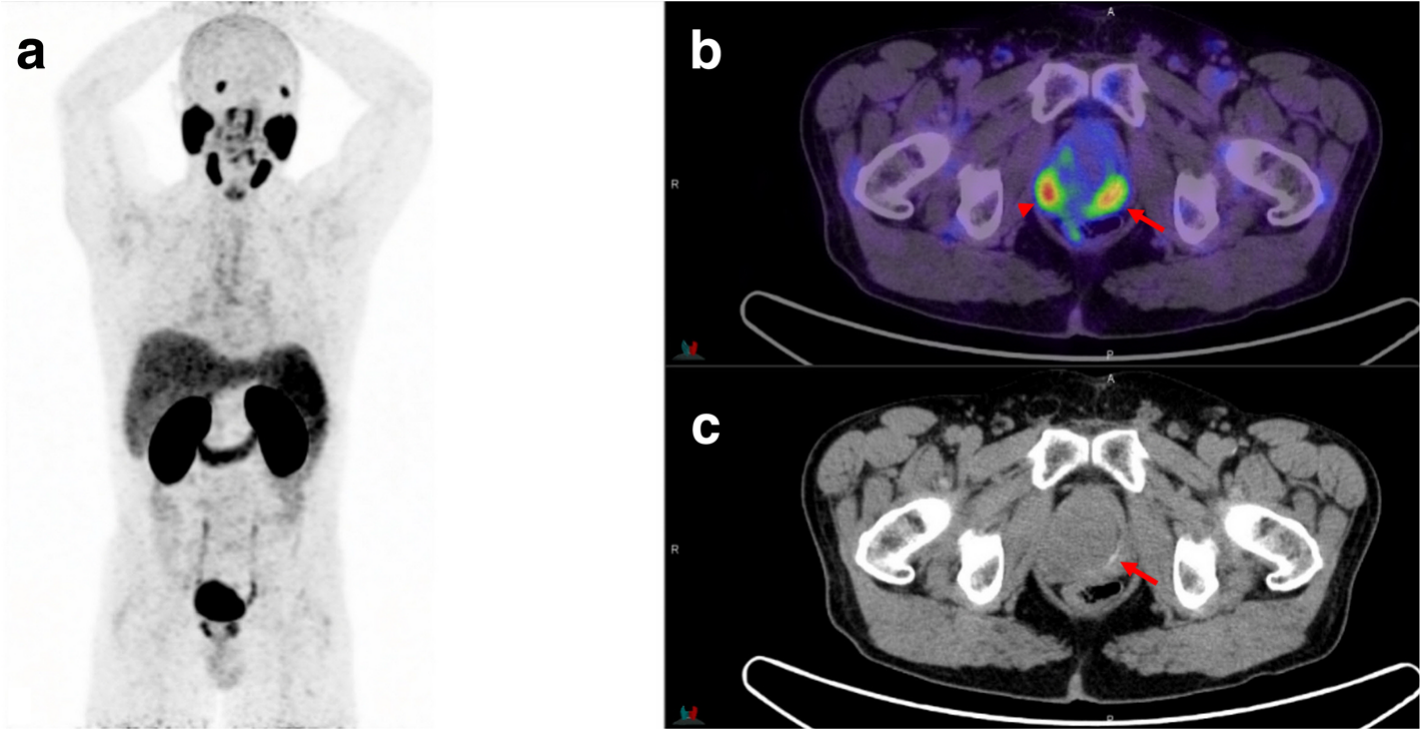
Sixty-year-old man for primary staging of prostate cancer and PSA 18 ng/L. Panel **a**. PSMA PET MIP image shows two areas of uptake on both sides of the prostate. Panel **b** and **c**. On PET/CT-U and CT-U images the left focus (arrow) corresponds to refluxed enhanced urine into prostate capsule and right focus (arrow head) is consistent with prostate cancer. Biopsies of both sides of prostate revealed only right sided malignancy

Overall 40 equivocal foci of activity were recorded in review of standalone PET images in 27/57 (47%) patients. On review of PET/CT-U, 11 foci in 10 patients (17.5%) corresponded to enhanced urine in the ureters, bladder, prostate bed, seminal vesicle or urethra. Of these, 3 foci (3 patients) were solitary sites of activity on PSMA imaging including two local and one nodal site. 4 foci (3 patients) were in different nodal fields, either contralateral pelvis or ipsilateral pelvis but above or below common iliac vessels bifurcation (figs. 3, 4, 5). In addition, in one patient in PS group, PSMA activity in one side of prostate was attributed to refluxed urinary activity in the prostate capsule which was subsequently confirmed by histopathology (fig. 6).

**Fig. 7:**
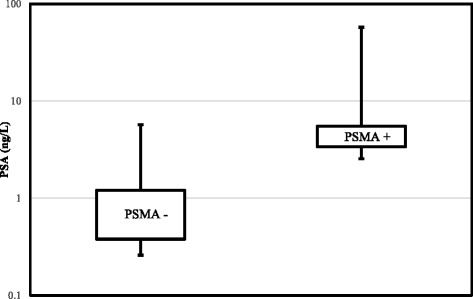
Logarithmic PSA values of patients with positive and negative PSMA PET

Table 1 shows the breakdown of the sites of recurrence in these patients. 75% (25/33) of patients with recurrence had at least one nodal metastasis. Overall 48 PSMA-expressing pelvic or para-aortic lymph nodes were detected in 25 patients. In 13 patients, there was only one positive lymph node. The parameters for ureters, lymph nodes and the distance between lymph nodes and ipsilateral ureters are detailed in the Table 2. No statistically significant difference is noted between the SUVmax of ureteric tracer activity and lymph nodes (*p* = 0.5). Low to moderate correlation was found between the SUVmax and short-axis diameter, long- axis diameter and the volume of the lymph nodes (*r* = 0.43, 0.5 and 0.4, respectively). The mean PSA value of the patients with positive scan was statistically higher than the patients with negative scan (*p* = 0.038) (fig. 7).

**Table 1:** Breakdown of sites of recurrence in the patients with positive PSMA scan

Number of patients	Local	Lymph node	Bone	Other (liver)
16		+		
4	+			
3	+	+		
3		+	+	
2	+	+	+	
1	+	+	+	+

Some patients had multiple sites of recurrence as indicated by “+” sign

**Table 2:** Parameters in patients with BCR

Ureteric SUVmax	Lymph node SUVmax	Short axis (mm)	Long axis (mm)	Volume (ml)	Distance to ureter (mm)
8.8 ± 6.5 (3.8–31.4)	9.5 ± 6.6 (2.8–31.2)	8.3 ± 4.2 (1.5–18.9)	12 ± 5.5 (4.2–23)	0.94 ± 1.3 (0.05–6)	12.6 ± 9.5 (2–41)

All values are in mean ± SD (range)

### CT-U

In subgroup of patients with BCR, standalone CT detected 12 lymph nodes in 8 patients. The mean short axis of these lymph nodes was 12.8 ± 3.5 mm (9–18.9). Overall CT reported metastatic disease in 11 (22%) patients, all of whom also had confirmatory PSMA PET finding. In addition, PSMA PET did not show increased uptake in CT-reported equivocal findings in 5 patients. These included 4 patients with osseous abnormalities which did not have all characterics of PC metastases and one with enlarged retroperitoneal lymph nodes, which were subsequently proven to be lymphoma. 54.5% (31/57) of studies showed opacification of entire bilateral ureters, 31.5% (18/57) patchy or unilateral, 12% (7/57) proximal ureters and 2%(1/57) distal ureters. The mean distance between PSMA avid lymph nodes and opacified ureters, bladder, prostate bed, urethra or seminal vesicles was 12.6 ± 9.5 mm (range 2–41).

## Discussion

An important characteristic of prostate cancer is the expression of PSMA, which makes the tumors ideal targets for functional imaging [16–18]. ^68^Ga-PSMA-11 specifically binds to PSMA and an increasing number of studies suggest that PSMA PET/CT imaging is useful in detection of PC lesions [11, 12]. In a recent meta-analysis involving 1309 patients, detection rate for the PSA categories 0–0.2, 0.2–1, 1–2, and >2 ng/ml were 42%, 58%, 76%, and 95% scans, respectively [14]. Therefore, due to the very high positive predictive value and high detection rate even at low PSA values, PSMA PET could be considered as the standard of reference in BCR.

There is, however, significant ^68^Ga-PSMA-11 accumulation in the ureters and urinary bladder due to radiotracer excretion through kidneys [19]. This poses significant challenge in the assessment of the small lymph nodes in the proximity of the ureters or small local recurrence in peri-vesical region, prostate bed and urethra, particularly when potentially distorted by intervention. In our facility, we have adopted a new protocol using a modified CT-U with PET/CT imaging. In this approach, the CT was acquired at the time of ureteric and bladder opacification followed by PET acquisition and replaces the non-contrast or portal venous phase CT that is typically used for anatomical correlation and attenuation correction of PET data. In our experience, this technique is practical and could potentially assist in the final interpretation of about 17.5% of studies by localizing the focal tracer activity to the contrast enhanced urine. Especially, in three studies interpretation was changed from positive on PET to negative on PET/CT-U for site of recurrence. Multiple imaging protocols have been used to overcome this issue, which is not unique to ^68^Ga-PSMA imaging. Kamel et al. adopted a protocol including hydration and forced diuresis before FDG PET imaging for staging and restaging of urogenital malignancies such as bladder cancer. In this study, there was good visualization of the primary bladder cancer [20]. Rauscher et al. proposed injection of diuretic at the time of tracer injection to reduce artefacts associated with high tracer activity in the urinary collecting system and bladder [21]. This approach could also be added to our protocol, however would be more technically demanding with especial attention to the patient’s hydration status. Kabasakal et al. assessed the value of early pelvic imaging in the PSMA PET imaging in a retrospective study. PET/CT images of the pelvis were performed at 5 min followed by whole body images at 60 min post tracer injection. Comparison between early and late pelvic images revealed no difference in the number of lesions. Due to lack of bladder activity in early images, assessment of primary tumor and local lesions were easier but was potentially compromised by significantly lower lesional uptake in early images [22]. Furthermore, fitting multiple time-point imaging into the schedule of a busy PET department is difficult while our protocol provide one time point imaging. However, it should be noted, as we have not performed head to head comparison between PET/CT-U and PET/CT protocol, incremental value of the PET/CT-U or diagnostic advantage of this approach could not inferred from our data.

When imaging the ureter with CT-U, it can be difficult to achieve complete opacification and adequate distention with a single excretory phase [23]. We also experienced the same issue as only 54% of the CT-U images showed complete opacification of the entire length of bilateral ureters. Several techniques have been proposed to address this concern, such as oral or intravenous hydration; intravenous furosemide; use of abdominal compression devices; prone patient positioning; and additional delayed phase imaging but none have been universally effective in practice [23]. In addition, incorporating these techniques in PET/CT protocol can be impractical or potentially harmful but oral hydration remains the simplest approach. However, in our experience, even if the ureters do not completely opacify, CT-U helps in better delineation of the ureteric course. In addition, in the absence of corresponding focal contrast hold-up, an equivocal focal PSMA activity would be more likely interpreted as a lymph node (fig. 4, panel C).

Local recurrence is most frequently located near the vesico-urethral anastomosis or less commonly in the retained seminal vesicles, posterior bladder wall, recto-vesical region and at the resection site of the vas deferens [24]. Multi parametric(mp) MRI of the prostate can distinguish prostate cancer recurrence from residual healthy glandular tissue, post-treatment changes including scar/fibrosis or granulation tissue [25]. In a study by Freitag et al., mpMRI was compared to PSMA PET/MRI and PET/CT in detection of local recurrence. In this study, 50% of the patients with local recurrence on mpMRI were missed by PET and detection of local recurrence using the PET-component was significantly influenced by proximity to the bladder [26]. We have also observed pooling of the urinary activity in the proximal part of urethra, penile urethra and refluxed urine into the retained seminal vesicles particularly following prostatectomy (fig. 5). Even prior to prostatectomy, we have observed refluxed urinary contrast in the seminal vesicles or prostate capsule (fig. 6). These potentially pose significant challenges in differentiating local PSMA-avid disease from urinary activity. In our experience, contrast opacification of the bladder, bladder neck and urethra is, perhaps, the major advantage of this protocol in discriminating urinary activity from local recurrence. Evolving fluorinated PSMA agents with little or no early urinary excretion, however, could overcome this challenge [27].

In this study, standard dose CT was acquired for PSMA PET/CT-U protocol. The mean calculated effective radiation dose to patients form this imaging protocol was 18 mSv (16-20 mSv). CT acquisition during PET/CT imaging is often performed for a variety of purposes including diagnosis, anatomic localization of the PET images and attenuation correction of the PET images. CT for diagnosis purposes (110-200mAs) imparts more radiation dose (effective dose of 11-20 mSv) than CT for anatomic localization (30-60mAs) with effective radiation dose of 3-6 mSv [28, 29]. Therefore, if CT is performed only for anatomic localization of the PET images radiation often could be reduced by 50–80% [28–30]. In our study, stand-alone diagnostic CT detected metastatic disease in only 30% (11/33) of patients. It is unclear whether standard dose CT would have additional diagnostic value and this protocol could potentially be used with low dose CT.

A typical CT-U protocol has three phases including an initial unenhanced acquisition, nephrographic phase 90–100 seconds following administration of contrast agent and pyelographic phase images 5–15 minutes after contrast administration [15]. This is an excellent technique for the assessment of urothelial malignances. In this study, however, for the purpose of the ureteric and bladder opacification, only pyelogram phase of this imaging protocol has been acquired and referred to CT-U. In addition, we administered only 50 ml of contrast agent in this protocol whereas in the standard technique 100-150 ml of the nonionic contrast is commonly used [15]. A modification to this protocol is also possible by dual-bolus intravenous contrast administration with simultaneous nephrographic phase (portal-venous enhancement) and pyelographic phase in a single CT acquisition. Notwithstanding small risk of allergic reaction has to be considered. However, only two patients were not able to undergo this protocol due to prior contrast allergy or impaired renal function.

It has been reported that up to 80% of metastatic lymph nodes in PC have a short-axis diameter less than 7 mm [8, 9]. In a retrospective study of 21 patients Giesel et al. reported the mean short axis of 5.8 mm for the PSMA positive lymph nodes [31]. However, in our study, the mean short axis of PSMA positive lymph nodes was slightly higher at 8.3 mm. Nonetheless, this demonstrates the incremental value of the PMSA PET/CT over CT alone for assessment of BCR.

In our study, the overall detection rate for the any site of recurrence or metastasis was 66%. Afshar-Oromieh et al. reported the detection rate of 86.6% for PSMA PET in a retrospective study of 37 patients with BCR. The lower detection rate in our study could potentially be explained by the lower mean PSA levels of 2.0 ng/ml compared to 11.1 ng/ml [32]. This also may reflect referral of patients with BCR for investigation by PSMA PET with lower PSA levels and slower PSA kinetics than in prior series, based on the encouraging results recently presented in the literature.

This study has certain limitations. Firstly, there was no comparison group to demonstrate additional value of PET/CT-U to non-enhanced PET/CT. We instituted the CT-U protocol because of the uncertainty we experienced using non-contrast CT with PSMA PET. Our experience in the transition from a non-contrast to a CT-U protocol, however, is consistent with the findings in this study. The data provided, however, cannot quantify the incremental value of CT-U compared to unenhanced or standard portal venous phase CT. Lack of pathologic confirmation of lesions detected by ^68^Ga-PSMA PET/CT imaging or follow-up imaging is another limitation. It was not possible to obtain histologic verification because of ethical and practical reasons. However, given high positive predicative value of PSMA PET, this is becoming widely accepted as the standard of imaging.

## Conclusions

PSMA PET/CT-U is a practical protocol that can help in the interpretation these studies by distinguishing focal urinary activity in the ureters, bladder, prostate bed, seminal vesicles and urethra from low volume PC.

## Additional file

Additional file 1
**Table S1** Characteristics and modality based results in BCR patients. **Table S2** Characteristics and modality based results in PS patients. (DOCX 15 kb)

## Abbreviations

BCR: Biochemical relapse
CT: Computed tomography
CT-U: CT urography
eGFR: Estimated glomerular filtration rate
FWHM: Full-width at half-maximum
GS: Gleason score
mp: Multi parametric
MRI: Magnetic resonance
OSEM: Ordered subset expectation maximization
PC: Prostate cancer
PET: Positron emission tomography
PS: Primary staging
PSA: Prostate-specific antigen
PSMA: Prostate-specific membrane antigen
SUVmax: Maximum standardized uptake value

## References

[1] Siegel R, Ma J, Zou Z, Jemal A (2014). Cancer statistics, 2014. CA Cancer J Clin.

[2] Heidenreich A, Bastian PJ, Bellmunt J, Bolla M, Joniau S, van der Kwast T, Mason M, Matveev V, Wiegel T, Zattoni F (2014). EAU guidelines on prostate cancer. Part II: treatment of advanced, relapsing, and castration-resistant prostate cancer. Eur Urol.

[3] Hodolic M (2011). Role of (18)F-choline PET/CT in evaluation of patients with prostate carcinoma. Radiol Oncol.

[4] Horvat A, Kovač V, Strojan P (2009). Radiotherapy in palliative treatment of painful bone metastases. Radiol Oncol.

[5] Heidenreich A, Bellmunt J, Bolla M, Joniau S, Mason M, Matveev V, Mottet N, Schmid HP, van der Kwast T, Wiegel T (2011). EAU guidelines on prostate cancer. Part 1: screening, diagnosis, and treatment of clinically localised disease. Eur Urol.

[6] Cornford P, Bellmunt J, Bolla M, Briers E, De Santis M, Gross T, Henry AM, Joniau S, Lam TB, Mason MD (2017). EAU-ESTRO-SIOG guidelines on prostate cancer. Part II: treatment of relapsing, metastatic, and castration-resistant prostate cancer. Eur Urol.

[7] Kosuri S, Akhtar NH, Smith M, Osborne JR, Tagawa ST (2012). Review of salvage therapy for biochemically recurrent prostate cancer: the role of imaging and rationale for systemic salvage targeted anti-prostate-specific membrane antigen radioimmunotherapy. Adv Urol.

[8] Hovels AM, Heesakkers RA, Adang EM, Jager GJ, Strum S, Hoogeveen YL, Severens JL, Barentsz JO (2008). The diagnostic accuracy of CT and MRI in the staging of pelvic lymph nodes in patients with prostate cancer: a meta-analysis. Clin Radiol.

[9] Flanigan RC, McKay TC, Olson M, Shankey TV, Pyle J, Waters WB (1996). Limited efficacy of preoperative computed tomographic scanning for the evaluation of lymph node metastasis in patients before radical prostatectomy. Urology.

[10] Eder M, Eisenhut M, Babich J, Haberkorn U (2013). PSMA as a target for radiolabelled small molecules. Eur J Nucl Med Mol Imaging.

[11] Afshar-Oromieh A, Haberkorn U, Eder M, Eisenhut M, Zechmann CM (2012). **[**68Ga]gallium-labelled PSMA ligand as superior PET tracer for the diagnosis of prostate cancer: comparison with 18F-FECH. Eur J Nucl Med Mol Imaging.

[12] Afshar-Oromieh A, Malcher A, Eder M, Eisenhut M, Linhart HG, Hadaschik BA, Holland-Letz T, Giesel FL, Kratochwil C, Haufe S (2013). PET imaging with a [68Ga]gallium-labelled PSMA ligand for the diagnosis of prostate cancer: biodistribution in humans and first evaluation of tumour lesions. Eur J Nucl Med Mol Imaging.

[13] Eiber M, Maurer T, Beer AJ, Souvatzoglou M, Weirich G, Kubler H: Prospective evaluation of PSMA-PET imaging for preoperative lymph node staging in prostate cancer *J Nucl Med* 2014 55 (Supplement 1).

[14] Perera M, Papa N, Christidis D, Wetherell D, Hofman MS, Murphy DG, Bolton D, Lawrentschuk N (2016). Sensitivity, specificity, and predictors of positive 68Ga-prostate-specific membrane antigen positron emission tomography in advanced prostate cancer: a systematic review and meta-analysis. Eur Urol.

[15] O'Connor OJ, Fitzgerald E, Maher MM (2010). Imaging of hematuria. AJR Am J Roentgenol.

[16] Reinhardt JM, Maleike D, Pluim JPW, Fabel M, Tetzlaff R, von Tengg-Kobligk H, Heimann T, Meinzer H-P, Wolf I (2008). Lymph node segmentation on CT images by a shape model guided deformable surface methodh. Proc SPIE.

[17] Sterzing F, Fiedler H, Stefanova M, Afshar-Oromieh A, Kratochwil C, Debus J, Haberkorn U, Giesel F (2014). Impact of 68Ga-PSMA PET/CT in Staging of Prostate Cancer Patient Prior to Radiation Therapy. International Journal of Radiation Oncology*Biology*Physics.

[18] Wright GL, Grob BM, Haley C, Grossman K, Newhall K, Petrylak D, Troyer J, Konchuba A, Schellhammer PF, Moriarty R (1996). Upregulation of prostate-specific membrane antigen after androgen-deprivation therapy. Urology.

[19] Silver DA, Pellicer I, Fair WR, Heston WD, Cordon-Cardo C (1997). Prostate-specific membrane antigen expression in normal and malignant human tissues. Clin Cancer Res.

[20] Kamel EM, Jichlinski P, Prior JO, Meuwly JY, Delaloye JF, Vaucher L, Malterre J, Castaldo S, Leisinger HJ, Delaloye AB (2006). Forced diuresis improves the diagnostic accuracy of 18F-FDG PET in abdominopelvic malignancies. J Nucl Med.

[21] Rauscher I, Maurer T, Fendler WP, Sommer WH, Schwaiger M, Eiber M (2016). **(**68)Ga-PSMA ligand PET/CT in patients with prostate cancer: how we review and report. Cancer Imaging.

[22] Kabasakal L, Demirci E, Ocak M, Akyel R, Nematyazar J, Aygun A, Halac M, Talat Z, Araman A (2015). Evaluation of PSMA PET/CT imaging using a 68Ga-HBED-CC ligand in patients with prostate cancer and the value of early pelvic imaging. Nucl Med Commun.

[23] Potenta SE, D'Agostino R, Sternberg KM, Tatsumi K, Perusse K, Urography CT (2015). For evaluation of the ureter. Radiographics.

[24] Cirillo S, Petracchini M, Scotti L, Gallo T, Macera A, Bona MC, Ortega C, Gabriele P, Regge D (2009). Endorectal magnetic resonance imaging at 1.5 tesla to assess local recurrence following radical prostatectomy using T2-weighted and contrast-enhanced imaging. Eur Radiol.

[25] Panebianco V, Barchetti F, Grompone MD, Colarieti A, Salvo V, Cardone G, Catalano C. Magnetic resonance imaging for localization of prostate cancer in the setting of biochemical recurrence. Urol Oncol. 2016;34:303-310. 10.1016/j.urolonc.2016.01.004 27012939

[26] Freitag MT, Radtke JP, Afshar-Oromieh A, Roethke MC, Hadaschik BA, Gleave M, Bonekamp D, Kopka K, Eder M, Heusser T (2017). Local recurrence of prostate cancer after radical prostatectomy is at risk to be missed in 68Ga-PSMA-11-PET of PET/CT and PET/MRI: comparison with mpMRI integrated in simultaneous PET/MRI. Eur J Nucl Med Mol Imaging.

[27] Giesel FL, Hadaschik B, Cardinale J, Radtke J, Vinsensia M, Lehnert W, Kesch C, Tolstov Y, Singer S, Grabe N (2017). F-18 labelled PSMA-1007: biodistribution, radiation dosimetry and histopathological validation of tumor lesions in prostate cancer patients. Eur J Nucl Med Mol Imaging.

[28] Brix G, Lechel U, Glatting G, Ziegler SI, Munzing W, Muller SP, Beyer T (2005). Radiation exposure of patients undergoing whole-body dual-modality 18F-FDG PET/CT examinations. J Nucl Med.

[29] Alessio AM, Kinahan PE, Manchanda V, Ghioni V, Aldape L, Parisi MT (2009). Weight-based, low-dose pediatric whole-body PET/CT protocols. J Nucl Med.

[30] Gelfand MJ, Lemen LC (2007). PET/CT and SPECT/CT dosimetry in children: the challenge to the pediatric imager. Semin Nucl Med.

[31] Giesel FL, Fiedler H, Stefanova M, Sterzing F, Rius M, Kopka K, Moltz JH, Afshar-Oromieh A, Choyke PL, Haberkorn U, Kratochwil C. PSMA PET/CT with Glu-urea-Lys-(Ahx)-[(6)(8)Ga(HBED-CC)] versus 3D CTvolumetric lymph node assessment in recurrent prostate cancer. Eur J Nucl Med Mol Imaging. 2015;42:1794-1800. 10.1007/s00259-015-3106-6 PMC4589548 26162799

[32] Afshar-Oromieh A, Zechmann CM, Malcher A, Eder M, Eisenhut M, Linhart HG, Holland-Letz T, Hadaschik BA, Giesel FL, Debus J, Haberkorn U. Comparison of PET imaging with a (68)Ga-labelled PSMA ligand and (18)F-choline-based PET/CT for the diagnosis of recurrent prostate cancer. Eur J Nucl Med Mol Imaging. 2014;41:11–20. 10.1007/s00259-013-2525-5 PMC3843747 24072344

