# Supplementary material for: 68Ga PSMA-11 PET with CT urography protocol in the initial staging and biochemical relapse of prostate cancer
Source: Cancer Imaging. 2017 Dec 21;17:31. doi: 10.1186/s40644-017-0133-5 (PMC5740783; doi:10.1186/s40644-017-0133-5)
Supplement: Additional file 1 — Table S1 Characteristics and modality based results in BCR patients. Table S2 Characteristics and modality based results in PS patients. (DOCX 15 kb) [file 40644_2017_133_MOESM1_ESM.docx]

| **Patient** | **Gleason Score** | **PSA** | **Any modality** | **PSMA PET** | **CT-U** | **PSMA PET/CT-U** |
| --- | --- | --- | --- | --- | --- | --- |
| 1 | 9 | 0.06 | Negative | Negative | Negative | Negative |
| 2 | 7 | 0.1 | Negative | Negative | Negative | Negative |
| 3 | 7 | 0.11 | Negative | Negative | Negative | Negative |
| 4 | 6 | 0.15 | Positive | Positive | Negative | Positive |
| 5 | 7 | 0.16 | Positive | Positive | Negative | Positive |
| 6 | 7 | 0.17 | Negative | Negative | Negative | Negative |
| 7 | 7 | 0.18 | Negative | Negative | Negative | Negative |
| 8 | 7 | 0.2 | Negative | Negative | Negative | Negative |
| 9 | 7 | 0.2 | Negative | Negative | Negative | Negative |
| 10 | 9 | 0.27 | Negative | Negative | Negative | Negative |
| 11 | 7 | 0.28 | Positive | Positive | Negative | Positive |
| 12 | 7 | 0.3 | Positive | Positive | Negative | Positive |
| 13 | 9 | 0.32 | Positive | Positive | Negative | Positive |
| 14 | 6 | 0.38 | Negative | Negative | Negative | Negative |
| 15 | 7 | 0.39 | Positive | Positive | Negative | Positive |
| 16 | 7 | 0.67 | Negative | Negative | Negative | Negative |
| 17 | 9 | 0.82 | Positive | Positive | Negative | Positive |
| 18 | 7 | 0.93 | Negative | Negative | Negative | Negative |
| 19 | 7 | 0.98 | Positive | Positive | Negative | Positive |
| 20 | 7 | 1 | Positive | Positive | Negative | Positive |
| 21 | 8 | 1.2 | Positive | Positive | Negative | Negative |
| 22 | 9 | 1.2 | Negative | Negative | Negative | Negative |
| 23 | 7 | 1.3 | Positive | Positive | Negative | Positive |
| 24 | 8 | 1.5 | Positive | Positive | Positive | Positive |
| 25 | 7 | 1.77 | Positive | Positive | Negative | Positive |
| 26 | 7 | 2 | Positive | Positive | Negative | Positive |
| 27 | 7 | 2.04 | Negative | Negative | Negative | Negative |
| 28 | 7 | 2.37 | Positive | Positive | positive | Positive |
| 29 | 7 | 2.5 | positive | positive | positive | Negative |
| 30 | 7 | 2.7 | Positive | Positive | Negative | Positive |
| 31 | 9 | 2.8 | Negative | Negative | Negative | Negative |
| 32 | 6 | 3.27 | Positive | Positive | Negative | Positive |
| 33 | 7 | 3.4 | Positive | Positive | Negative | Positive |
| 34 | 9 | 3.4 | Positive | Positive | Negative | Positive |
| 35 | 7 | 3.5 | Positive | Positive | Negative | Positive |
| 36 | 7 | 3.6 | Positive | Positive | Negative | Positive |
| 37 | 7 | 4.1 | Positive | Positive | Negative | Positive |
| 38 | 7 | 4.5 | Positive | Positive | positive | Positive |
| 39 | 9 | 4.8 | Positive | Positive | Negative | Positive |
| 40 | 9 | 4.8 | Positive | Positive | positive | Positive |
| 41 | 7 | 5.5 | Positive | Positive | positive | Positive |
| 42 | 7 | 5.7 | Positive | Positive | positive | Positive |
| 43 | 7 | 5.7 | positive | positive | Negative | Negative |
| 44 | 7 | 6.5 | Positive | Positive | Negative | Positive |
| 45 | 6 | 7.7 | Positive | Positive | Negative | Positive |
| 46 | 9 | 9.2 | Positive | Positive | positive | Positive |
| 47 | 7 | 10.3 | Positive | Positive | positive | positive |
| 48 | 9 | 23.9 | Positive | Positive | Negative | Positive |
| 49 | 7 | 56 | Positive | Positive | positive | Positive |
| 50 | 9 | 57.3 | Positive | Positive | Positive | Positive |

Table S1 Characteristics and modality based results in BCR patients

| **Patients** | **Gleason Score** | **PSA** | **Any modality** | **PSMA PET** | **CT-U** | **PSMA PET/CT-U** |
| --- | --- | --- | --- | --- | --- | --- |
| 1 | 7 | 6.8 | Negative | Negative | Negative | Negative |
| 2 | 10 | 9.1 | Negative | Negative | Negative | Negative |
| 3 | 7 | 18 | Negative | Negative | Negative | Negative |
| 4 | 7 | 18 | Negative | Negative | Negative | Negative |
| 5 | 7 | 20.1 | Negative | Negative | Negative | Negative |
| 6 | 7 | 40.1 | Positive | Positive | Negative | Positive |
| 7 | 9 | 100 | Negative | Negative | Negative | Negative |

Table S2 Characteristics and modality based results in PS patients
